# A simple surgical mask modification to pass N95 respirator-equivalent fit testing standards during the COVID-19 pandemic

**DOI:** 10.1371/journal.pone.0272834

**Published:** 2022-08-24

**Authors:** Agnes Z. Dardas, Viviana M. Serra Lopez, Lauren M. Boden, Daniel J. Gittings, Kevin Heym, Emily Koerber, Taras Grosh, Jaimo Ahn

**Affiliations:** 1 Department of Orthopaedic Surgery, University of Pennsylvania, Philadelphia, Pennsylvania, United States of America; 2 Orthopaedic Specialty Institute, Orange, California, United States of America; 3 Environmental Hygiene, University of Pennsylvania Health System, Philadelphia, Pennsylvania, United States of America; 4 Department of Anesthesiology and Critical Care, University of Pennsylvania, Philadelphia, Pennsylvania, United States of America; 5 Department of Orthopaedic Surgery, University of Michigan, Ann Arbor, Michigan, United States of America; University Hospital Heidelberg: UniversitatsKlinikum Heidelberg, GERMANY

## Abstract

**Background:**

The COVID-19 pandemic has infected hundreds of millions of people resulting in millions of deaths worldwide. While N95 respirators remain the gold standard as personal protective equipment, they are resource-intensive to produce and obtain. Surgical masks, easier to produce and obtain, filter ≥95% submicron particles but are less protective due to a lack of seal around a user’s face. This study tested the ability of a simple surgical mask modification using rubber bands to create a seal against particle exposure that would pass N95 standards.

**Methods and findings:**

Forty healthcare workers underwent TSI PortaCount mask fit testing using an ASTM Level 1 surgical mask modified with rubber bands. Fit Factor was determined after testing four standard OSHA N95 fit testing scenarios. Performance of the properly-modified surgical mask was compared to that of a poorly-modified surgical mask, an unmodified standard surgical mask, and an N95 respirator. Thirty-one of forty (78%) healthcare workers passed Fit Factor testing using a properly-modified mask. The Fit Factor success rate significantly improved by subsequent test date (p = 0.043), but was not associated with any other participant characteristics. The average Fit Factor score for the properly-modified mask was 151 (SD 65.2), a significantly better fit than the unmodified mask score of 3.8 (SD 3.1, p<0.001) and the poorly-modified mask score of 24.6 (SD 48.4, p<0.001) but significantly lower than a properly fitted N95 score of 199 (SD 4.5, p<0.001).do.

**Conclusions:**

Rubber bands, a low-cost and easily-accessible modification, can improve the seal and protective ability of a standard surgical mask to the level of an N95 respirator. This could mitigate N95 respirator shortages worldwide and provide individuals in under-resourced regions a practical means for increased personal respiratory protection.

## Introduction

The COVID-19 pandemic caused by Severe Acute Respiratory Syndrome Coronavirus 2 (SARS-CoV-2) has infected over 526 million individuals and caused more than 6.2 million deaths worldwide as of the end of May 2022 [1]. The financial and emotional tolls have been incalculable with ramifications still unknown. In the setting of droplet and aerosol-transmitted diseases, higher degrees of respiratory isolation through distance or physical barriers protect against infection [2]. While masks are a common type of personal protective equipment (PPE) used in healthcare and the community, N95 respirators represent the gold standard as PPE against air-borne illnesses. N95 respirators, however, are more resource-intensive to produce and obtain than surgical masks, as revealed during the SARS-CoV-2 pandemic. In March 2020, when China, the highest mask producer, increased production by a factor of 20 to 200 million masks per day, it was only able to produce 600,000 N95 respirators [3].

To achieve N95 designation by the US National Institute for Occupational Safety and Health (NIOSH), N95 respirators must demonstrate a minimum filtration efficiency of ≥95% of particle sizes of 0.3μm [4]. Surgical masks, on the other hand, must meet ASTM International F2100 standards that mandate filtration efficiency of ≥ 95% at 0.1μm [5]. Surgical masks do not meet N95 standards primarily because they lack a seal around a wearer’s face, allowing particles to bypass the filter and enter peripherally [6, 7].

The creation of a seal around a standard surgical mask is hypothesized to protect the wearer and others against particle exposure by isolating air exchange via mask filtration. The group at www.fixthemask.com first proposed the use of rubber bands to create such a seal, calling on independent validations of their hypothesis [8]. Proof of concept was confirmed by Runde et al in a small group of 11 subjects who used a PortaCount machine and 3 rubber bands tied around a surgical mask using a paper clip and a face shield [9]. However, this previous study had an inadequate sample size to validate their results according to standard respiratory fit panels [10]. Therefore, this prospective cross-sectional cohort study aimed to determine if an even simpler modification of a surgical mask, using just 2 readily-available 8” rubber bands, could improve the seal of a surgical mask to pass N95 standards according to standard respiratory fit panel testing methods.

## Methods

### Testing details

#### Pilot testing

**Fig 1:**
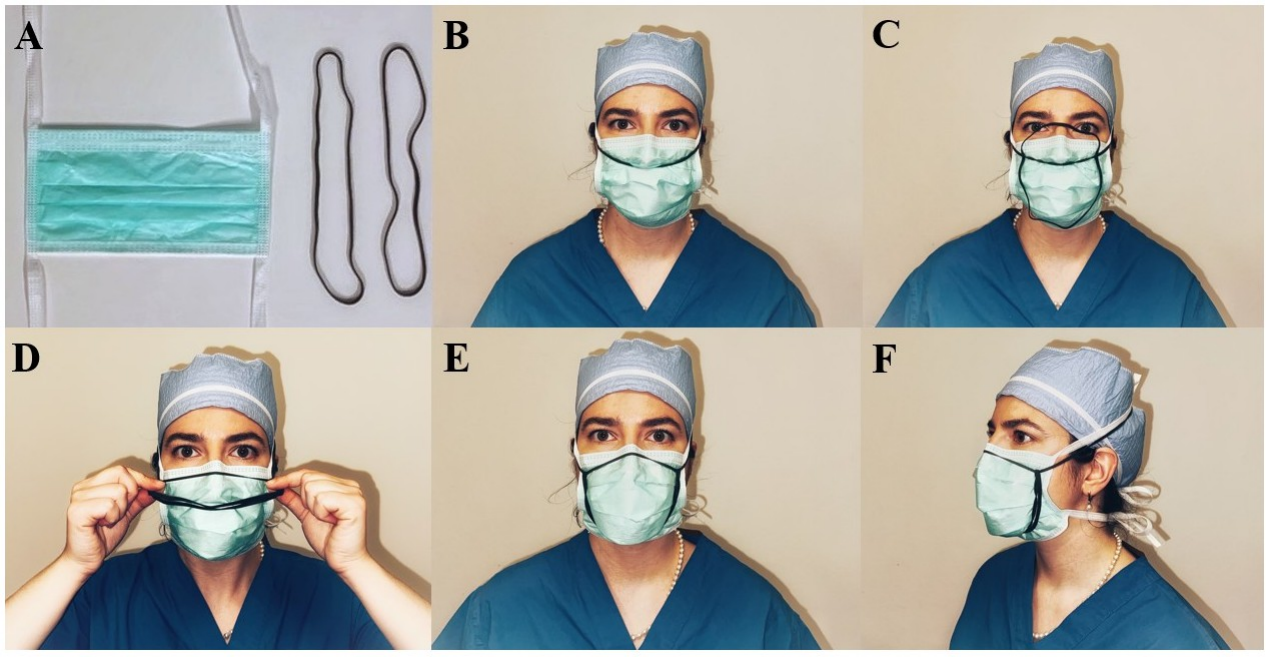
Demonstration of the assembly of the modified mask. Photographs of the simple modification demonstrating an individual properly donning the modified mask. (A) Modified mask components consist of a standard ASTM Level 1 surgical mask and two 8” rubber bands. (B) While wearing an ASTM Level 1 surgical mask which has been adjusted to fit along the bridge of the nose, apply one 8” rubber band along the crown of the head and place the front of the rubber band under the nose. (C) Take another 8” rubber band and apply it perpendicularly under the first rubber band so that two loops are formed above and below the first rubber band. (D) Shift the first rubber band so that it is over the bridge of the nose and fold the second rubber band in half on itself along the first rubber band on the horizontal axis. € Place the second rubber band along the cheeks and under the chin. Adjust both rubber bands as needed to achieve a full seal as shown in this anterior view of the final construct. (F) Lateral view of the final construct.

In April 2020, five study investigators (AZD, VSL, LB, DJG, JA) underwent fit testing to determine the feasibility of mask modification and establish baseline particle testing of NIOSH-certified N95 respirators (3M 8210, 3M 1860) and ASTM Level 1-certified surgical masks (Precept 15211), the hospital’s standardly-available PPE. Quantitative testing was performed using a NIOSH-certified TSI PortaCount 8048 Fit Tester with associated particle generator. Mask modification was performed using two rubber bands (Coopay, large) (Fig 1).

#### Subject selection

In May 2020, adult subjects at a single tertiary care health system in the United States were prospectively recruited, enrolled, and underwent mask fit testing using a TSI PortaCount 8048 machine. Participants completed an electronic screening survey to pass inclusion and exclusion criteria. Inclusion criteria included adults ≥ 18 years of age who were approved for clinical work within the health system, able to read and understand English, and provide informed consent. Exclusion criteria included pregnancy, latex allergy, facial hair or other features that would normally preclude N95 respirator wear, prior COVID-19 positive testing, and symptoms concerning for respiratory illness including cough, shortness of breath, and elevated temperature. If screening criteria were met, potential participants were directed to an online informed consent form, consistent with COVID-19 social distancing protocols.

#### Study procedures

Subjects meeting screening criteria who provided written informed consent were invited to a single 15-minute in-person testing session with study personnel. During this session, participants were provided with a new disposable ASTM Level 1 Precept 15211 surgical mask (standard for the health system) and two 8” rubber bands (Coopay, large; Amazon.com). They were trained to properly modify their surgical masks using the two rubber bands placed in a standardized fashion (Fig 1). Four PortaCount fit testing exercises–bending, talking, head side to side, head up and down—were performed twice for each subject, once with the properly-modified technique and once with a poorly-modified technique. The properly-modified technique placed the rubber bands over the crown of the subject’s head, bridge of the nose, around the cheeks, and under the chin within the boundaries of the surgical mask (Fig 1). The poorly-modified technique required subjects to move the rubber bands beyond the extent of the mask’s confines with the intent to create a leak and simulate a hasty incorrectly-placed mask scenario (Fig 1). Subjects were closely monitored for adverse events during testing. Basic demographics were collected at the end of testing and individual results were shared with the subject. A follow-up survey was electronically distributed after testing to determine the comfort and security of the mask modification.

This study was conducted after full committee review and approval by the University of Pennsylvania Perelman School of Medicine’s Investigational Review Board which also reviewed and approved an Investigational Device Exemption application as low risk. All participants provided written informed consent before taking part in this study. The individual in this manuscript whose image was used to demonstrate the mask modification also provided written informed consent as outlined in PLOS’s consent form to publish their case details. Mask training and PortaCount testing were performed by the same investigators for all subjects (AZD and EK) utilizing the same PortaCount 8048 machine. All survey answers and fit testing results were collected and managed securely in a REDCap database [11].

### Statistical analysis

#### Outcomes

The primary outcome was pass or fail of the modified mask. This was determined using the overall PortaCount Fit Factor which was reported on a scale of 1–200+, with 100 being the OSHA passing threshold for N95 respirators. Secondary outcomes were comparative analyses of the quantitative PortaCount performance score between four cohorts (unmodified masks, properly-modified masks, poorly-modified masks, and N95 respirators). Additional outcomes included the subjective feeling of comfort and security while wearing a modified mask.

#### Sample size

A sample size of 40 with a threshold passing rate of 30 (75%) is sufficient in minimizing type I and type II errors for respirator fit testing panels [10]. If the null hypothesis is that a properly-modified mask remains as ineffective as an unmodified surgical mask, then passing the respirator fit test rejects the null hypothesis. In Landsittel et al, a passing rate of 75% in a sample size of 40 subjects was calculated to minimize a type I error, falsely passing a respirator test, to 3.5% and to minimize a type II error, falsely failing a respirator test, to 16.1%, well within the usual statistical thresholds of 5% and 20% respectively [10]. A sample size of 40 also exceeded the minimum sample size of 26 calculated during an *a priori* sample size analysis to detect a Fit Factor difference of 50 at 90% power with a two-sided alpha of 0.05 based on the variances from pilot testing data (WinPepi software). Enrollment was stopped once sample size was reached to limit mask destruction and non-clinical interactions.

#### Analyses

Subjects’ baseline characteristics, the primary outcome measure, and survey questions were analyzed using descriptive measures with non-parametric statistics such as median and interquartile range (IQR). For the purposes of statistical analyses, the maximum Fit Factor of 200+ was recoded to 201. Secondary outcome comparisons were performed using Wilcoxon Rank-Sum and Mann-Whitney U tests for dependent and independent-sample comparisons respectively. Categorical comparisons were made using chi-square and Fischer’s exact tests. Statistical significance was set at p<0.05 without explicit adjustment for multiple comparisons for secondary outcomes. Analyses were performed with SPSS software, version 26 (IBM Corp).

## Results

### Participant characteristics

**Fig 2:**
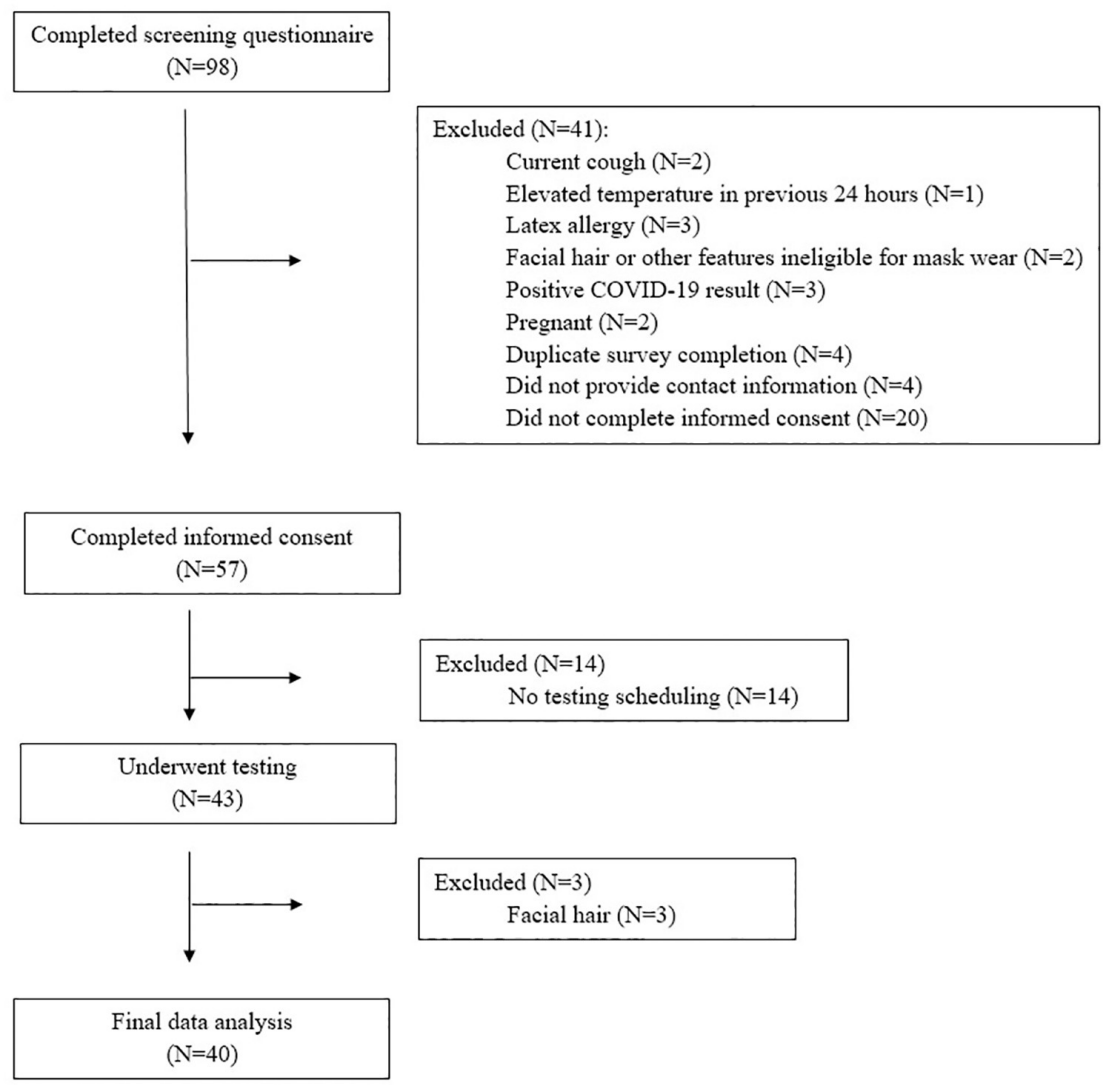
Study flow diagram from screening to data analysis.

In May 2020, 43 adult subjects underwent mask fit testing using the modified surgical mask, of which 40 were included in the final data analysis; three were excluded due to facial hair noted by study personnel but not reported by the subjects during the screening questionnaire (Fig 2). The median age of participants was 39 years old (IQR 32–55), with a range of 25–68 years. 58% were female. 73% reported no prior N95 fit testing issues, 20% reported prior difficulty passing N95 fit tests, and 8% were never tested despite working in a healthcare setting.

### Comparison to N95 passing criteria

**Fig 3:**
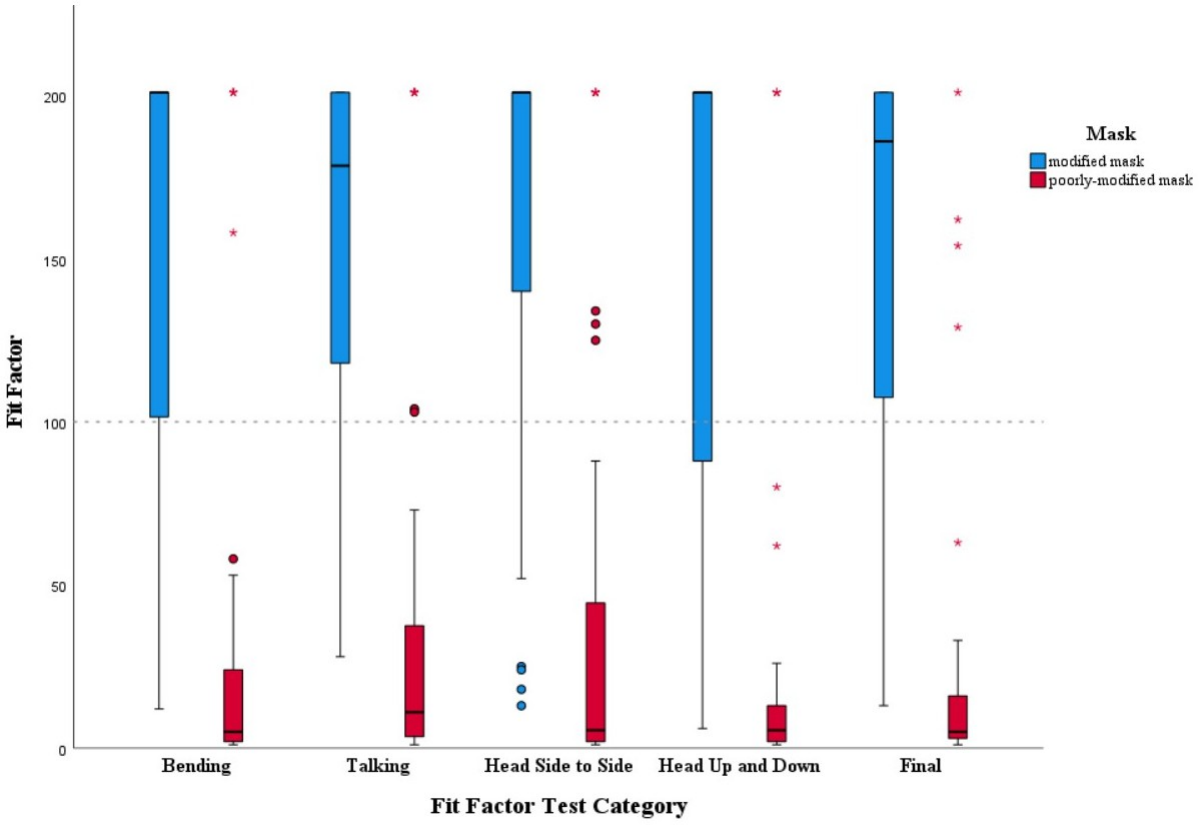
A comparison of appropriately modified and poorly-modified mask performance using quantitative PortaCount fit testing. Fit Factor subscores of bending, talking, head side to side, and head up and down, as well as Final PortaCount Fit Factor scores are presented as boxplots. Boxplots are structured such that the horizontal line within the box indicates the median, the vertical length of the box is the IQR, and the whiskers demonstrate the minimum and maximum values excluding the outliers which are circles and stars. Circles are outliers that are 1.5 times the IQR. Stars are outliers that are 3 times the IQR. In each pairing, the properly modified group is on the left and the poorly-modified group is on the right. The dotted horizontal line through the 100 y-axis marker is the passing threshold.

Of the 40 health care workers included in the final analysis, 31 of the participants wearing modified surgical masks scored 100 or greater on the Final PortaCount Fit Factor, a PortaCount-generated composite score based on four standard subscores of bending, talking, head side to side, head up and down (Fig 3). This surpassed the reported standard passing criteria of N95 respirators [10]. Of the 31 subjects that passed, 27 (87%) tested in the highest quarter of possible scores, at a level of 150–200+. The four other scores were 107, 108, 127, and 141. Of the nine participants whose modified surgical masks failed Fit Factor testing, 78% failed with a score in the lowest quarter of possible scores, at a level of 1–49. Two of the failures were within 26 points of passing.

### Poorly-modified mask performance

90% of participants failed mask fit testing with the poorly-modified mask (median 5, IQR 3–16) (Fig 3). 35 of the 36 (97%) participants who failed scored 1–49; 3 of the 4 (75%) who passed scored 150–200+.

### Mask comparisons

**Fig 4:**
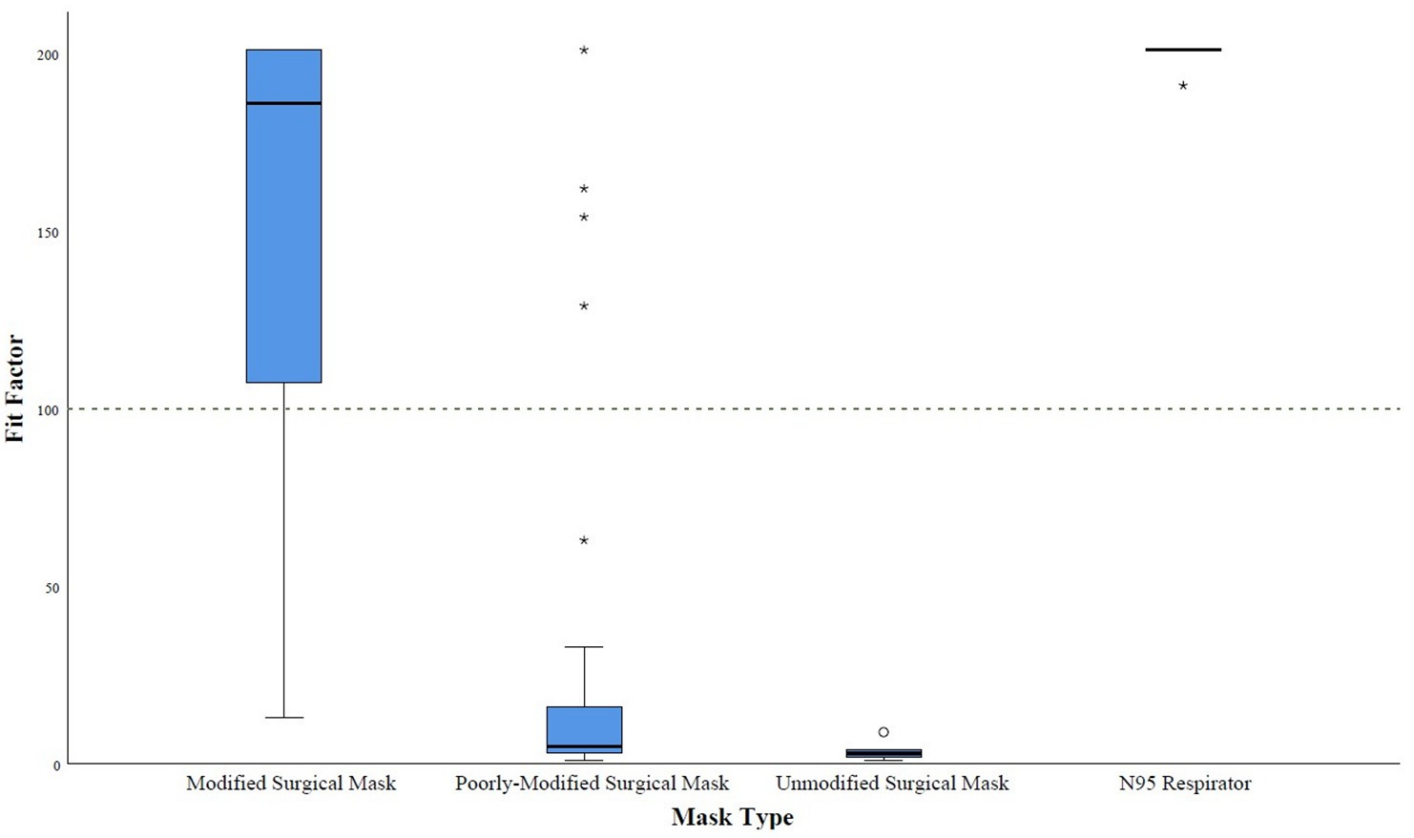
Comparative summative fit performance between appropriately modified, poorly-modified, unmodified surgical masks, and N95 respirators based on quantitative PortaCount testing. Boxplots demonstrate the horizontal line within the box as the median, the vertical length of the box as the IQR, and the whiskers as the minimum and maximum values excluding outliers which are circles and stars. Circles are outliers that are 1.5 times the IQR. Stars are outliers that are 3 times the IQR. The dotted horizontal line through the 100 y-axis marker is the passing threshold.

With respect to Fit Factor, while the modified surgical mask’s median score of 186 (IQR 107–201) performed statistically significantly better than the OSHA passing threshold of 100 (p <0.001), this same score was significantly lower than the N95 respirator’s pilot testing median score of 201 (IQR 196–201) (p = 0.023) (Fig 4). The modified surgical mask’s median score of 186 (IQR 107–201) outperformed the poorly-modified surgical mask’s median score of 5 (IQR 3–16) (p<0.001) and the unmodified surgical mask’s median score of 3 (IQR 1.5–6.5) (p<0.001) (Fig 4). The poorly-modified surgical mask was not significantly better than the unmodified surgical mask (p = 0.194) (Fig 4).

### Comfort and secure fit of the modified mask

**Fig 5:**
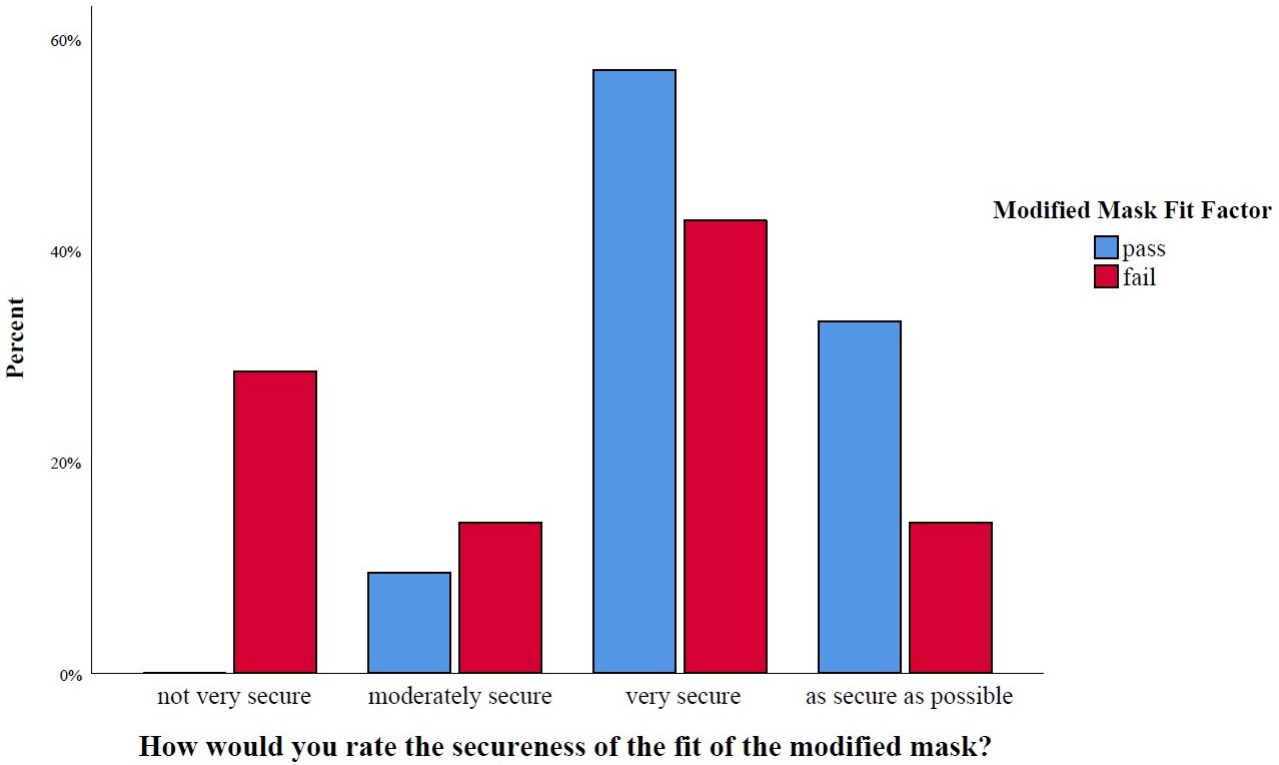
Subjective secure fit of the modified mask compared to the surgical mask stratified by outcome.

23 of 28 (82%) participants who responded to the post-testing questionnaire felt that the modified mask was secure. 100% of the participants whose modified mask passed gave the modified mask a secure rating, compared to only 71% of the participants whose modified mask did not pass N95 criteria (p = 0.056) (Fig 5).

**Fig 6:**
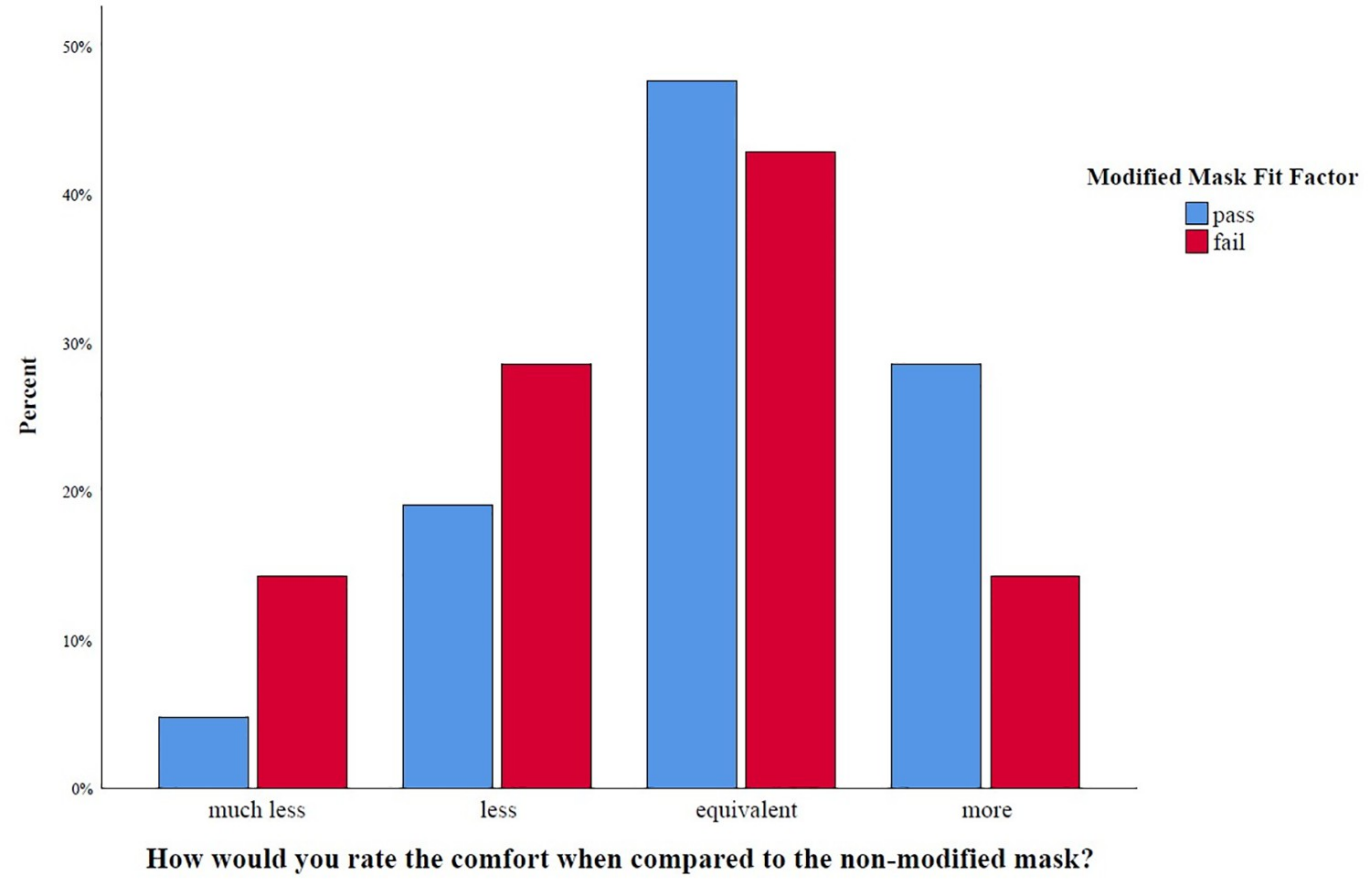
Subjective comfort of the modified mask compared to the surgical mask stratified by outcome.

71% of survey responders rated the modified mask as or more comfortable to an unmodified surgical mask. 76% of those who passed ranked it as equivalent or more comfortable, compared to 57% of those who failed (Fig 6) (p = 0.371).

### Factors associated with primary outcome

**Fig 7:**
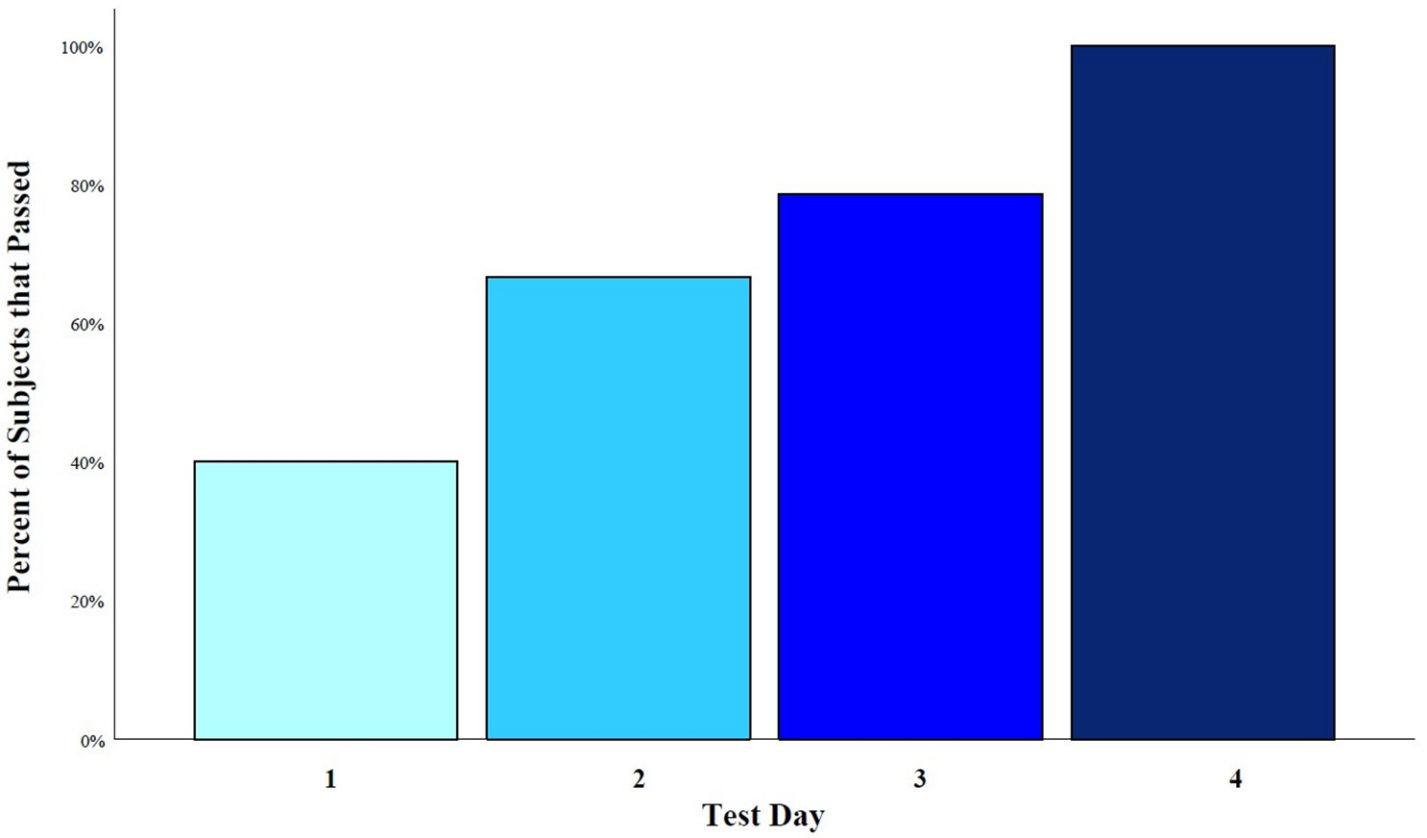
Fit Factor passing rate by test day.

Age, gender, prior fit testing issues, reported comfort, and perceived security were not associated with passing Fit Factor testing for the modified mask (p-values > 0.05). Only the test date was associated with increased success rate of the modified mask (p = 0.043), with each subsequent test date resulting in a higher passing rate; 40% of 5 participants passed on day 1, 67% of 9 participants passed on day 2, 79% of 14 participants passed on day 3, and 100% of 12 participants passed on day 4 (Fig 7). There were no statistically significant differences between test dates with respect to age, gender, and prior fit testing issues that could otherwise account for this difference.

### Adverse events

No adverse events occurred during fit testing sessions with any mask.

## Discussion

We found that modifying the surgical mask with rubber bands passed the same OSHA Fit Factor testing criteria used for N95 respirator testing. The performance of the modified mask was independent of factors such as age and gender, but positively associated with subsequent test date, indicating a learning curve in teaching participants proper rubber band placement and subjective seal-check performance. While the results of the Fit Factor by activity were not blinded to participants and study personnel, once Fit Testing began no adjustments to the mask were allowed to minimize bias from mid-test results. The quantitative testing results were also not operator-dependent nor modifiable.

Several studies indicate that there is no “one-N95-fits-all” mask on the market, with N95 passing rate variability [12–16]. In a US healthcare worker study of 1,271 participants, 95% of male subjects passed Fit Factor testing compared to 85% of female testers [13]. In a separate US study testing three common N95 respirators, 28% of participants did not pass a fit test with any mask [14]. Chinese and Korean populations have shown worse performance with 0–45% passing rates on Chinese fit test panels [15] and 16–48% in Korean military hospital workers [16]. Differences in face dimensions are thought to account for this, highlighting the need for a more customized approach.

Despite the mixed clinical data, a properly-fitted N95 respirator remains the clinical gold standard, resulting in shortages of N95 respirators during the COVID-19 pandemic. PPE shortages inspired new innovations of pre-existing supplies such as the creation of mask braces, highlighting the feasibility of modifying a surgical mask with rubber bands to improve filtration efficiency [9, 17, 18]. This data provided additional compelling rationale for our study to determine whether improved filtration efficiency specifically in ASTM Level 1 masks could pass N95 level testing thresholds.

Interestingly, 10% of participants passed Fit Factor testing with a poorly-modified surgical mask, a variation with an incomplete seal made by improperly placing the rubber bands beyond the confines of the surgical mask. This was intended to control the variable of rubber band placement, indicating that it was not rubber band placement on a surgical mask alone, but rather proper placement, that created the seal. This unexpected finding can likely be attributed to the rubber bands pressing on the soft tissue of the face to meet the surgical mask and create a better seal on the sides of the face and chin. Despite these outliers, the poorly-modified surgical mask did not test significantly differently from the unmodified surgical mask.

### Limitations

The primary limitations of this study was the *in vitro* testing paradigm, lack of facial anthropometric data, and learning curve associated with teaching others to don the modification. Further studies are needed to determine compliance, adverse events, and effectiveness against aerosolized diseases when worn for a prolonged period. While several studies address facial dimensions, our protocol intentionally limited contact between subjects and testers to maintain social distancing, sacrificing measuring data that would require closer contact. This simulated the more common fit testing scenario where a healthcare worker is tested without prior knowledge of facial dimensions.

We were limited in our control cohort size (n = 5), resulting in a comparatively larger experimental number (n = 40). The primary reason for this was practical and ethical: because quantitative testing is destructive, we could not justify the destruction of 40 additional N95 masks for testing alone in the setting of a pandemic.

Lastly, the learning curve associated with teaching others how to apply the modification was an unexpected finding but critical nonetheless. While further studies are required to compare multiple instructors’ success rates with different students over time, our preliminary data suggests that multiple training sessions are needed before users are fully effective in teaching others.

### Strengths

Strengths of our study include quantitative fit testing to assess the primary outcome, an appropriate sample size validated in the literature, and the demonstration of a low-cost and easily-adaptable intervention. The TSI PortaCount is one of the industry standards for OSHA N95 fit testing, with similar false passing rates but significantly lower false failing rates compared to the Bitrex and Saccharin tests [19]. Our sample size of 40 with a pre-determined passing level of 30 corresponds with less than 10% type II errors and 5% type I errors [10]. Lastly, the intervention in this study costs a fraction of the price of an N95 respirator. Whereas a single 3M 1860 N95 respirator from McKesson, a leading industry distributor, can be $1.06, a single ASTM level 1 surgical mask costs $0.19 through the same distributor and 2 8” rubber bands cost $0.23 on Amazon [20–22]. This results in a $0.42 cost for a modified surgical mask, less than half the price of an N95 respirator. In addition, the differences in price are likely magnified downstream as an incremental increase in cost translates to disproportionately increased difficulty in obtaining scarce respirators in a sellers’ market. While it is beyond the scope of this study to accurately assess the availability of rubber bands in under-resourced regions of the world, it is likely easier to redistribute rubber bands and surgical masks compared to N95 respirators from appropriately resourced regions to under-resourced regions in the world due to their increased production and reduced cost.

## Conclusions

In conclusion, this study prospectively and rigorously tested a simple rubber-band modification of ASTM level 1 surgical masks relative to industry-standard N95 respirator criteria. While this modification is not meant to replace N95 respirators when they are available, it may allow for the conservation of N95 respirators in lower-risk settings and supplement them when they are relatively scarce, such as during a pandemic or in countries with limited resources and access. While the inspiration for this study and its data are meant to help protect against COVID-19, it could be generalized to protect from any aerosolized disease including but not limited to influenza, MERS, SARS, tuberculosis, and anthrax.
